# Biological effects of dosing aerobic exercise and neuromuscular electrical stimulation in rats

**DOI:** 10.1038/s41598-017-11260-7

**Published:** 2017-09-07

**Authors:** Stefania Dalise, Loredana Cavalli, Harmanvir Ghuman, Brendon Wahlberg, Madeline Gerwig, Carmelo Chisari, Fabrisia Ambrosio, Michel Modo

**Affiliations:** 1 University of Pittsburgh, McGowan Institute for Regenerative Medicine, Pittsburgh, Pennsylvania USA; 2 Department of Bioengineering, Pittsburgh, Pennsylvania USA; 3 Department of Radiology, Pittsburgh, Pennsylvania USA; 4 Department of Neuroscience, Pittsburgh, Pennsylvania USA; 5 Department of Physical Medicine and Rehabilitation, Pittsburgh, Pennsylvania USA; 6 University Hospital of Pisa, Department of Neuroscience, Unit of Neurorehabilitation, Pisa, Italy

## Abstract

Aerobic exercise (AE) and non-aerobic neuromuscular electric stimulation (NMES) are common interventions used in physical therapy. We explored the dose-dependency (low, medium, high) of these interventions on biochemical factors, such as brain derived neurotrophic growth factor (BDNF), vascular endothelial growth factor-A (VEGF-A), insulin-like growth factor-1 (IGF-1) and Klotho, in the blood and brain of normal rats, as well as a treadmill-based maximum capacity test (MCT). A medium dose of AE produced the most improvement in MCT with dose-dependent changes in Klotho in the blood. A dose-dependent increase of BDNF was evident following completion of an NMES protocol, but there was no improvement in MCT performance. Gene expression in the hippocampus was increased after both AE and NMES, with IGF-1 being a signaling molecule that correlated with MCT performance in the AE conditions, but also highly correlated with VEGF-A and Klotho. Blood Klotho levels can serve as a biomarker of therapeutic dosing of AE, whereas IGF-1 is a key molecule coupled to gene expression of other molecules in the hippocampus. This approach provides a translatable paradigm to investigate the mode and mechanism of action of interventions employed in physical therapy that can improve our understanding of how these factors change under pathological conditions.

## Introduction

Aerobic exercise (AE) and neuromuscular electric stimulation (NMES) are common interventions used in physical therapy for peripheral nerve regeneration^1^, traumatic brain injury^2, 3^, stroke^4, 5^, spinal cord injuries^6^ and neurodegenerative disease. Optimization of these paradigms is essential to ensure that an appropriate dose is given to ensure therapeutic efficacy^7^, without causing overtraining or muscle injury^8^. A major challenge in a clinical setting is the determination and verification that a sufficient dose of therapy is delivered to promote optimal functional recovery^9, 10^. Evidence-based medicine is reliant on experimental results that guide the clinical decision making process to define appropriate therapeutic interventions, including physical therapy^11–13^. Establishing training characteristics and blood-borne biomarkers, which can be repeatedly sampled, provide avenues to validate existing interventions, as well as to optimize their use for individual subjects^14, 15^.

Minimally invasive biomarkers are key indicators of the impact that therapeutic interventions exert on cells, tissues and organ systems. Biomarkers should reflect therapeutic dosing, as well as changes in functional performance. In patients, blood provides a readily accessible source of biological material that can be longitudinally sampled to monitor changes in factors associated with performance improvement^14, 16, 17^. A major opportunity of blood-borne biomarkers lies in their potential for translation between animal and human studies^18, 19^. Studies in rodents provide more control over biological variables and a more stringent assessment of performance characteristics to tease out dose-dependency, but also more extensive mechanistic and invasive studies, including histological evaluations of muscle and brain tissue^20^. In the context of AE and NMES, biomarkers of particular interest suggested to be responsive to physical activity include: brain derived neurotrophic factor (BDNF), which is involved in neurogenesis, vascular endothelial growth factor (VEGF)-A involved in angiogenesis, and insulin-like growth factor-1 (IGF-1) promoting growth and maintenance of muscle^21, 22^. More recently, it has been suggested that Klotho, known for its anti-aging effects and neuroprotective activity^23–25^, may also be responsive to exercise^26, 27^.

As these biochemical factors follow a circadian rhythm^23, 28^, time of day is an important variable to consider while evaluating these biomarkers^29^. A circadian rhythm is also observed in behavioral activity^30^. Although rats are nocturnal animals and are naturally more active during the dark phase of the day^31^, standard training and testing is generally performed during the light condition^32^. A major confound during the dark cycle is the different amount of fatigue that animals display due to their natural activity^29, 33^. Training and testing during the light cycle hence provides a consistent baseline for animals to evaluate experimental interventions without fatigue as a confounding variable. Translation of biomarkers and behavioral measures to human subjects therefore needs to consider the influence of the light-dark cycle on these parameters, as even artificial lighting can affect performance and growth factors in humans^34^. Biological sex also influences the response to AE^35^, with male subjects exhibiting larger effects^35^.

In the current study, treadmill running and NMES were chosen as training paradigms, as these can be reliably administered in both rodents and human subjects to contrast aerobic and non-aerobic physical therapy interventions on the same organ systems. Treadmill running, as an AE paradigm, provides a well-established protocol to measure maximum exercise capacity in humans^36, 37^ and is an excellent functional measure in patients^38^. Treadmill-based maximum capacity testing (MCT) presents a unique opportunity to apply the same standardized paradigm in rodents^39^, providing an outcome measure with high face validity in the context of bench-to-bedside translation^40^. NMES also has high face validity for translation and affords a direct comparison of the biological effects of muscle stimulation in the absence of aerobic activity. NMES is an important intervention to reduce spasticity following a stroke^41^ and to enhance recovery of muscle strength and function in the case of severe functional impairments, such as paresis, that cannot sufficiently support gait to walk or run^5, 42, 43^. Contrasting the biological effects of these two interventions aimed at the same organ system (muscle, blood, brain) is therefore possible.

To establish a basic understanding of the biological and functional effects of AE and NMES, we investigated these two treatment paradigms in normal rats over a 4 week period, using MCT as the primary behavioral outcome measure. For each of these interventions, three different dosing levels and a control condition with no treatment provided a factorial design that afforded the definition of optimal dosing of therapy. Apart from controlling key variables, such as age and time of testing, a key advantage of performing animal studies is that other tissues, such as the brain, are also readily available for analysis and allow us to evaluate the correlation between changes in blood-borne biomarkers and gene expression in specific brain regions, such as the hippocampus. This study therefore provides a unique understanding of how dosing of two commonly utilized physical therapy interventions affects key biomarkers in normal subjects.

## Methods

### Experimental Design

**Figure 1:**
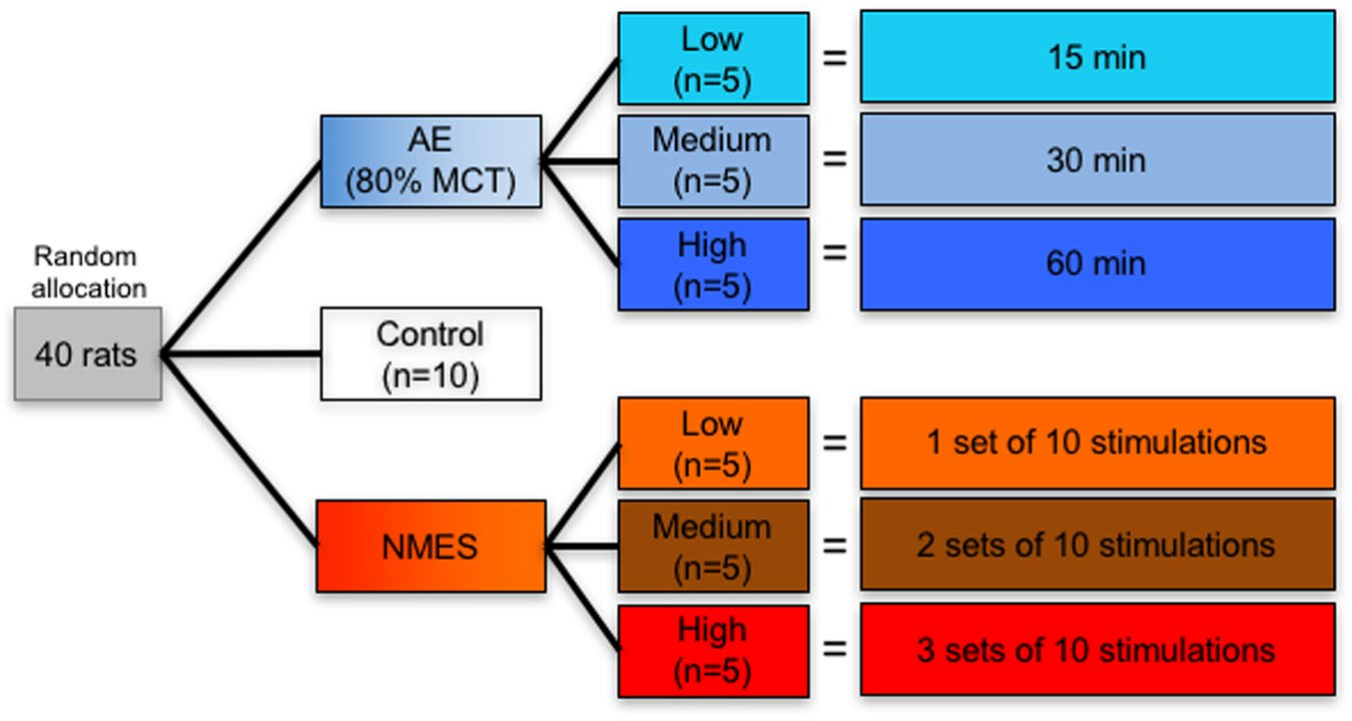
Experimental design and methodology. Animals were randomly allocated to 7 experimental conditions upon arrival. An untreated control group and two training paradigms consisting of aerobic exercise (AE) and neuromuscular electrical stimulation (NMES), each with 3 doses (low, medium, high), were compared.

All animal procedures complied with the US Animals Welfare Act (2010) and were approved by the University of Pittsburgh Institutional Animal Care and Use Committee (IACUC). Sprague-Dawley rats (male, n = 40, 12 weeks of age, 260 ± 15 g, Taconic Labs, USA) were maintained on a 12-hour light/dark schedule (lights on 06:00), with food and water available *ad libitum*. To avoid confounding effects of the estrous cycle on biomarkers and their relationship to performance^44^, only male rats were used here to establish a proof-of-principle dosing of AE and NMES and their impact on biomarkers. Animals were randomly assigned (using a random number sequence) into 3 treatment groups (Fig. 1): control, aerobic exercise (AE), and neuromuscular electrical stimulation (NMES). Using a 3 levels factorial design, the AE and NMES treatments were further divided into low, medium and high loading conditions. Animals’ weight and maximum capacity for exercise were evaluated at baseline (week 0) and after 4 weeks of treatment to provide a final outcome measure. All treatment and maximum capacity testing occurred between 09:00–16:00 to avoid the ambulatory period with the animals’ active period during the dark period (18:00–06:00).

### Maximum capacity test (MCT)

MCT of exercise performance in rats was performed, akin to the Bruce Protocol used in human subjects^36^. Although physiology-based (e.g. inhaled volume of oxygen, blood lactate) cardiopulmonary exercise testing is considered the gold standard for evaluating disorders affecting the cardiovascular, pulmonary, and/or skeletal muscle systems^45^, these measures are less relevant in the context of physical therapy, where a behavioral readout, such as the Bruce protocol, remains the most commonly used measure of maximum capacity for exercise^37^. Measurement of blood lactate levels to determine the lactate threshold (LT) indicating exhaustion, as a measure of maximum capacity^46^, is aimed at assessing endurance rather than peak effort^47^. Defining the LT requires the repeat sampling of blood, which induces a stress response affecting heart rate and corticosterone in animals^48, 49^. The LT was not used here to avoid potential confounds in blood biomarkers and behavioral read-outs. Although it is noted that a functional treadmill-based protocol is not adequate for athletic testing^37^, it provides a non-invasive and non-stressful mean to define dosing of AE in the context of physical therapy (see below). The MCT is a means to measure maximum capacity of exercise using a treadmill that can be applied in humans and rodents. Treadmill and wheel running are widely used in rodent experiments as therapeutic interventions, but there is currently a lack of a standardized testing procedure to assess the functional effects of these procedures. We therefore here aimed to mirror the principles set out in the Bruce Protocol to provide a standardized framework that can easily be implemented by different basic science laboratories, as well as in a translational setting.

For this, rats were first familiarized with the treadmill (AccuScan Instruments) to reduce the overall stress response to the new environment and to familiarize them with the equipment designed specifically for rodents (Supplementary Figure 1A). All animals were acclimatized to the 4-lanes motor-driven treadmill for 10 min/day at a rate of up to 10 m/min for two consecutive days. The following day, their maximum capacity for exercise was determined. To measure maximum capacity for exercise, all animals underwent treadmill running at 0° inclination, starting with a set speed of 5 m/min, and gradually increasing speed by 5 m/min every 3 minutes (Table 1). Each revolution of the belt is 47.6 cm with 10.5 revolutions occurring for a 5 m/min speed. Based on the time spent running, distance and speed are calculated. Animals readily ran on the treadmill. During the initial testing phase at low speed, animals occasionally fell back and required light prodding to ensure that they were running to their maximum capacity. Maximum capacity for exercise was reached if animals could no longer run on the treadmill belt and fell back to the empty space for 3 seconds or more. No electric shock was used to motivate running, as it was considered to provide a stressful association with the task.

**Table 1:** Rat maximum capacity testing (MCT) protocol.

Stage	Duration (min)	Time (min)	Speed (m/min)	Speed (km/h)	Distance (m)	Total distance (m)	Belt Revolutions
1	3	3	5	0.3	15	15	31.5
2	3	6	10	0.6	30	45	94.5
3	3	9	15	0.9	45	90	189.1
4	3	12	20	1.2	60	150	315.1
5	3	15	25	1.5	75	225	472.1
6	3	18	30	1.8	90	315	661.8
7	3	21	35	2.1	105	420	882.4
8	3	24	40	2.3	120	540	1134.5

### Aerobic exercise (AE)

To evaluate the effects of AE on MCT and biomarkers, animals were allocated to a low (15 min), medium (30 min) or high (60 min) running condition set at a speed of 80% of their maximal baseline activity, thereby accounting for individual differences in performance characteristics. An 80% level of MCT is often used in animal models as aerobic training to induce positive oxidative physiological adaptations^50^. The lactate threshold occurs at approximately 50–60% of the VO2max in untrained and 65–80% in trained rats^46^. A sub-maximal 80% level of MCT in a clinical setting is considered a vigorous intensity^51^. A single running session per day, 5 times/week (Mon.-Fri.), at 80% was used to determine which condition provided the most significant improvements over a 4 weeks training period.

### Neuro-muscular electrical stimulation (NMES)

To perform NMES in the rat^52, 53^, animals underwent general anesthesia using isofluorane (4% induction, 1.5–2% maintenance). Respiration was assessed regularly to monitor depth of anesthesia. The left forearm was shaved to expose the skin and provide a better conductance contact for electrodes to exert position-triggered NMES (Supplementary Figure 1B). A conductive gel was placed on biceps and triceps prior to placement of the surface electrode, which was connected to a stimulator (Empi 300 PV, St Paul, USA). The NMES protocol was performed 5 days/week (Mon.–Fri.) for 4 weeks. The NMES protocol consisted of a low (1 set), medium (2 sets) or high (3 sets) protocols of 10 contractions/set. The parameters used to stimulate the muscles were: 150 µs pulse duration, 50 Hz frequency, 5 s time on, 10 s time off, 0.5 s ramp and 0.5 s ramp down. A pulse intensity of 15.0 mA was applied to the biceps and triceps to achieve a flexion (Supplementary Figure 1C) or extension (Supplementary Figure 1D). The correct placement of the electrode on the biceps or triceps was confirmed when stimulation elicited an elbow flexion or extension (Supplementary Figure 2), respectively, of at least a 30° of range of motion in either direction (Supplementary Figure 1E), as measured using a goniometer (Supplementary Figure 1F). Stimulation sets were applied alternately on the biceps and triceps muscles.

### Sample harvesting

To evaluate the biological effects of AE and NMES, blood and brain were collected from all animals for further analysis. For this, animals were terminally anesthetized using an overdose of FatalPlus (Henry Schein). The chest cavity was exposed and blood was drawn directly from the right heart atrium into a syringe. Blood was transferred into an Eppendorf and left for at least 60 min at room temperature before further processing (see below). After collection of the blood sample, rats were perfused transcardially with ice-cold 0.9% saline followed by 4% paraformaldehyde (in 0.2 M PBS) to fix brain tissue prior to its removal from the skull. The excised brain was post-fixed overnight in 4% paraformaldehyde before being transferred to 30% sucrose (with sodium azide) for storage at 4 °C prior to tissue processing.

### Blood serum isolation and enzyme-linked immunosorbent assay (ELISA)

After collection of whole blood in a closed vial, blood was allowed to clot for 60 minutes by leaving it undisturbed at room temperature (21 °C). The coagulated blood was then discarded after centrifugation at 1000 g for 15 minutes. Supernatant blood serum was collected in a clean polypropylene tube (ThermoFischer). Serum samples were aliquoted and stored at −80 °C until analysis.

Brain-derived neurotrophic factor (BDNF, ERBDNF, Thermo scientific), Vascular endothelial growth factor-A (VEGF-A, RRV00, R&D), Insulin-like growth factor 1 (IGF-1, CSB-E04582r, CUSABIO) and Klotho (CSB-E14958r, CUSABIO) levels were determined by quantitative enzyme-linked immunosorbent assay (ELISA) kits. For this, all reagents and samples were brought to room temperature before use. 50–100 µL of standards and diluted serum samples (Dilution factors: 1x for VEGF, 2x for BDNF, 200x for IGF-1 and 1000x for Klotho; diluted in assay diluent) were added to wells and incubated at room temperature (VEGF and BDNF) or 37 °C (IGF-1 and Klotho) for 2 hours with gentle shaking. Assay diluent served as a zero standard (0 pg/mL) for background subtraction to construct a standard curve. For VEGF and BDNF assays, the solution was then discarded and the wells were washed four times with 400 µL of wash buffer before adding 100 µL of VEGF conjugate solution or BDNF biotinylated antibody and incubating for 1 hour at room temperature. For IGF-1 and Klotho assays, the sample solutions were discarded before directly proceeding (w/o washing) with the addition of a biotin-antibody for 1 hour at room temperature. Wells were washed 4x with buffer. For the VEGF assay, 100 µL of substrate solution was added to each well and incubated for 30 mins at room temperature before adding 50 µL of stop solution. For BDNF, IGF-1 and Klotho assays, an additional step consisted of adding 100 µL HRP-avidin to each well for 60 mins at 37 °C, before washing 5x with buffer. 90–100 µL of TMB substrate was then added in each well for 30 mins before adding the stop solution.

Wells were protected from light at all times after the addition of substrate solution. Optical density of each well was measured using an automated microplate reader (PowerWave 340, Bio-Tek) set to 450 nm (correction wavelength set to 540 nm) within 30 minutes of adding the stop solution. A standard curve was constructed by plotting the mean absorbance for each standard against the concentration to draw a best-fit curve through the points on the graph. If the samples were diluted, the concentration read from the standard curve was then multiplied by the dilution factor.

### mRNA isolation and quantitative PCR of hippocampal tissue

Histological sections (50 μm thickness) were cut on a cryostat (Leica) directly onto microscopic slides to preserve tissue morphology. Hippocampal tissue from the left hemisphere was segmented from 10 sections (originating 500 μm apart) using a scalpel. The RNeasy FFPE kit (Qiagen) was used to isolate total RNA, according to the kit protocol. Tissue sections were suspended in a 1 mL Eppendorf tube in kit PKD buffer, and treated with Proteinase K at 56 °C, for 30 min, and 80 °C for 20 min. The supernatant after centrifugation was treated with kit DNAse I for 15 min at room temp. Samples were diluted with RBC and ethanol before loading onto kit columns for centrifugation to bind RNA to the matrix. Columns were washed with RPE and RNA was eluted with kit elution buffer. RNA was stored at −80 °C and quantitated with a Qubit Fluorometer (ThermoFisher). A typical yield from ten tissue sections was 1–2 μg of RNA. cDNA samples were prepared from 500 ng RNA with the High-Capacity RNA-to-cDNA Kit (ThermoFisher).

Quantitative real-time polymerase chain reaction (qRT-PCR) was used for relative quantification of targets, which consisted of BDNF (#Rn02531967_s1 TaqMan Gene Expression Assay, Applied Biosystems), VEGFA (#Rn01511602_m1, Applied Biosystems), IGF1 (#Rn00710306_m1, Applied Biosystems) and KLOTHO (#Rn00580123_m1, Applied Biosystems). Expression levels of the target genes and reference gene β-actin (#Hs01060665_g1, Applied Biosystems) were assessed in duplicates using the TaqMan Fast Advanced Master Mix reagent (ThermoFisher) on a StepOne Real-Time PCR System (Applied Biosystems). Initial denaturation was conducted for 2 min at 50 °C and 20 s at 95 °C, followed by 40 cycles of 1 s at 95 °C and 20 s at 60 °C. A melt curve was generated for primer specificity from 60 °C to 95 °C at 1 °C intervals for 10 s. The threshold cycle (Ct) values of the target genes were averaged and expressed as fold change by dividing the Ct value of the target gene by the Ct value of the β-actin control before a log2 transform.

### Statistics

Graphing was performed in Prism version 7 (GraphPad). Statistical analyses and contour plots were calculated in Minitab version 17 (Minitab). For repeated measures of weight and MCT, a two-way factorial ANOVA was performed with one factor being time and another being intervention (i.e. AE versus NMES) with 3 levels each. For group comparisons of % change, ELISAs, and mRNA analysis, a 3 (interventions) × 3 (levels) factorial design ANOVA was performed, as all data was distributed normally with equal variance. Post-hoc testing using Sidak compared experimental conditions directly to controls. Pearson’s correlations were calculated as behavioral and biological measures were normally distributed. For contour plots, performance changes in MCT, as indicated by percent speed change, were plotted along the X axis for all plots. The Y axis defined the different levels of treatments administered to animals, ranging from no treatment (i.e. control condition) to a high treatment conditions for treadmill running (60 min) or NMES (30 stimulations). Dependent variables were plotted against these independent variables in 6 equal data ranges (bin sizes) to define contour lines that span the measured values and provide an overview of the interaction of independent variables on the dependent variables. The definition of optimal dosing levels based on both MCT performance and biomarkers is also feasible using this approach based on the gradients of measured variables (i.e. speed change on MCT and individual biomarkers).

## Results

### Aerobic exercise improves running performance

**Figure 2:**
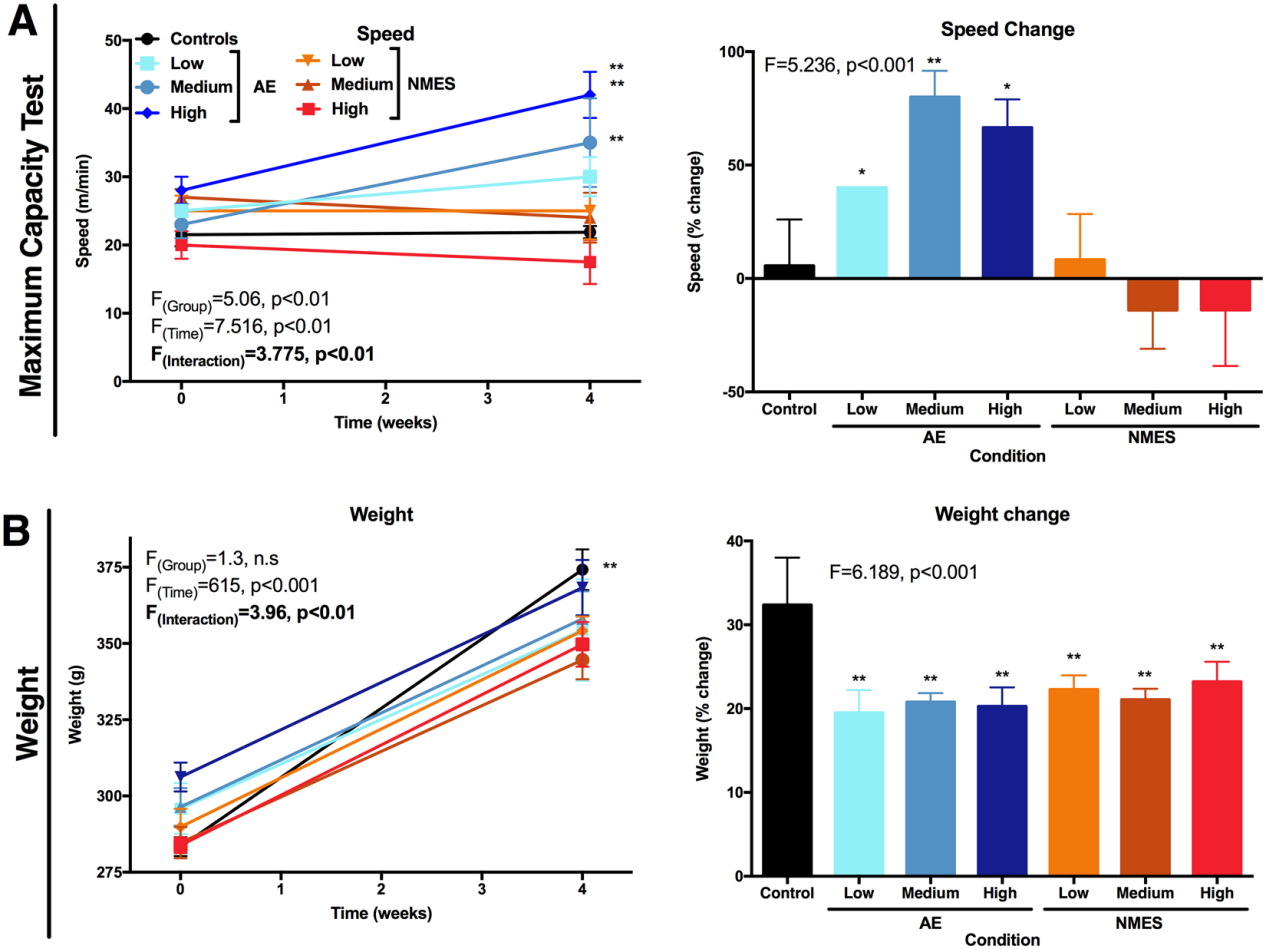
Maximum capacity testing (MCT) and body weight. (**A**) The MCT measured speed and time at baseline (week 0) and after 4 weeks. A percentage change was calculated between both time points to account for individual differences at the starting point. (**B**) Weight of animals was measured at baseline and the final time point (4 weeks) to determine if treadmill training was reducing weight gain in comparison to other conditions and hence could exert an indirect effect on MCT. Percentage change between both time points was calculated to account for individual differences at baseline. (*p < 0.05; **p < 0.01).

After random allocation to experimental groups, maximum capacity testing (MCT) was performed at baseline to establish basic performance characteristics of individual rats. All groups performed at a similar level between 15 and 30 m/min, with a mean performance of 24 m/min (Fig. 2A). No significant difference was evident at baseline between groups. Nevertheless, to account for individual performance differences in training, parameters for individual animals in the AE condition were adjusted to 80% of their baseline performance for dosing. Running speed in the untreated animals in the control group did not change over a four week period. After four weeks of training, all of the animals in the AE group ran faster than they did in pre-exercise tests (Fig. 2A). The low treadmill condition improved performance by 40% (p < 0.01), with the medium condition exhibiting the most improvement with 80% change (p < 0.01). In contrast, none of the NMES treatment conditions lead to improvements in MCT performance (Fig. 2A). The NMES high condition revealed a non-significant trend to perform worse by 14%. These results therefore indicate that treadmill training selectively improved MCT performance, with a medium dosing providing the most efficient paradigm.

As AE demands a metabolic effort compared to the other conditions, it is conceivable that this could affect weight gain and produce a confounding covariate of performance on MCT. Although control animals gained significantly (p < 0.01) more weight than animals receiving AE or NMES treatments, there was no significant difference in weight between the AE and NMES conditions (Fig. 2B). This, therefore, indicates that MCT performance differences between the AE and NMES conditions was not affected by weight gain.

### Klotho reflects a dose-dependency of AE and changes in performance

**Figure 3:**
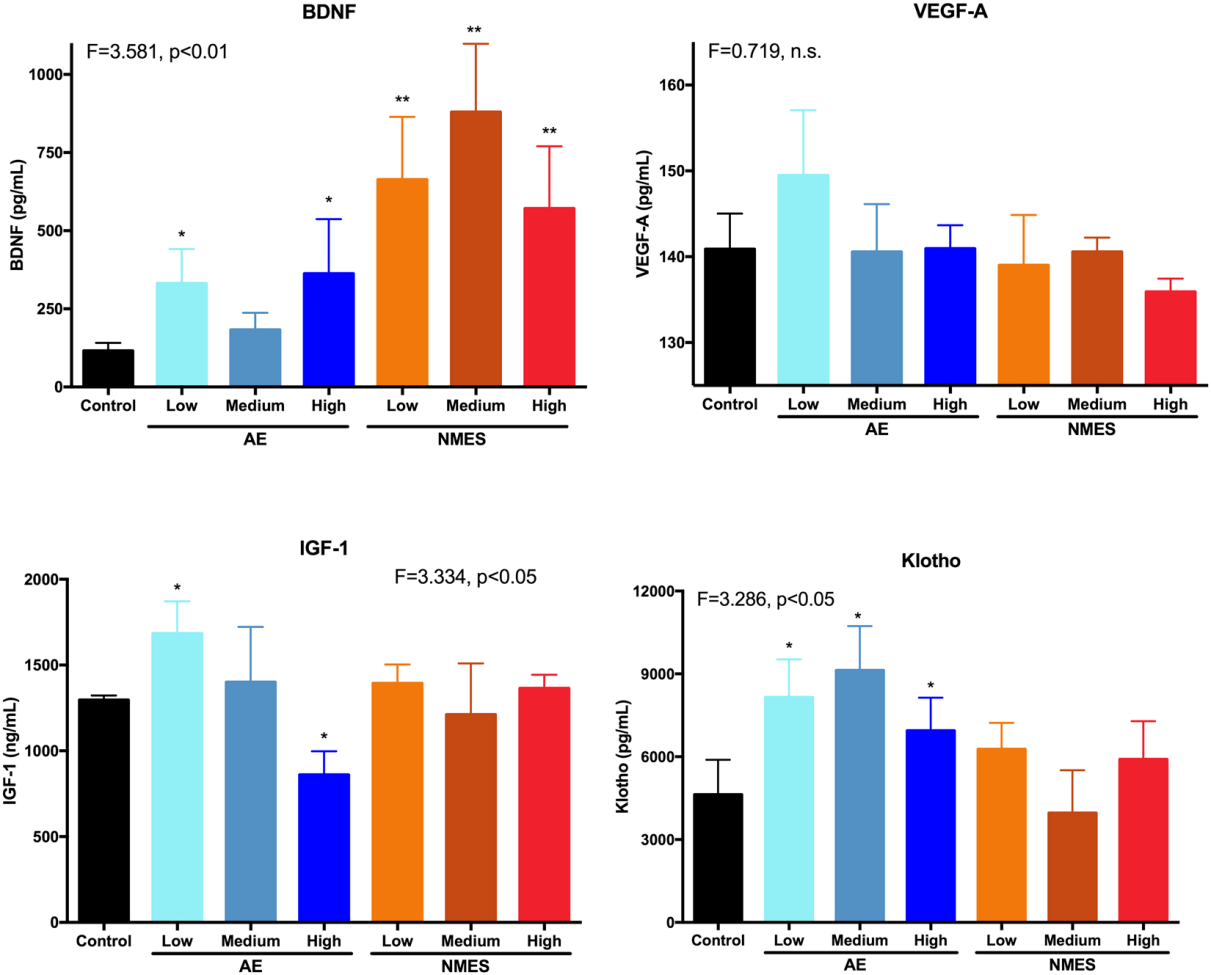
Comparison of systemic factors using enzyme-linked immunosorbent assays (ELISA). Brain-derived nerve growth factor (BDNF) was upregulated after both AE and NMES, with the most dramatic change evident after a medium dose of NMES resulting in an almost 8-fold increase. Little change was evident in VEGF-A, with only the low AE condition showing a minor non-significant 6% increase. The low AE condition also increased IGF-1 by 33% and showed an AE dose-dependent decrease, with the medium condition being equivalent to control levels. NMES did not affect IGF-1 levels. In contrast, Klotho revealed a significant AE dose-dependent modulation of levels with a medium dose of AE doubling the amount of Klotho in the blood compared to controls. NMES also produced an increase of Klotho for the low and high condition, but not for the medium dose. (*p < 0.05; **p < 0.01).

Measurement of biochemical factors in the blood at the final time point after 4 weeks of training revealed a differential regulation depending on treatment, as well as dosing. Apart of the medium AE condition, BDNF was significantly (p < 0.05) increased in all treatment conditions (Fig. 3). NMES conditions increased blood levels more than two-fold, as compared to AE. The medium NMES condition achieved the highest BDNF level in blood, 7.6x the level of controls. In contrast, VEGF-A did not significantly increase or decrease in any condition, when compared to controls (Fig. 3). IGF-1 levels for controls and NMES were equivalent (Fig. 3). AE produced a significant (p < 0.05) dosing effect with low dosing of AE increasing (23%) IGF-1 levels and a high dose decreasing (34%) IGF-1 (Fig. 3). Klotho levels were only significantly (p < 0.05) increased for AE conditions, with the highest level being produced by the medium AE condition (Fig. 3). NMES did not significantly affect Klotho levels in the blood.

**Figure 4:**
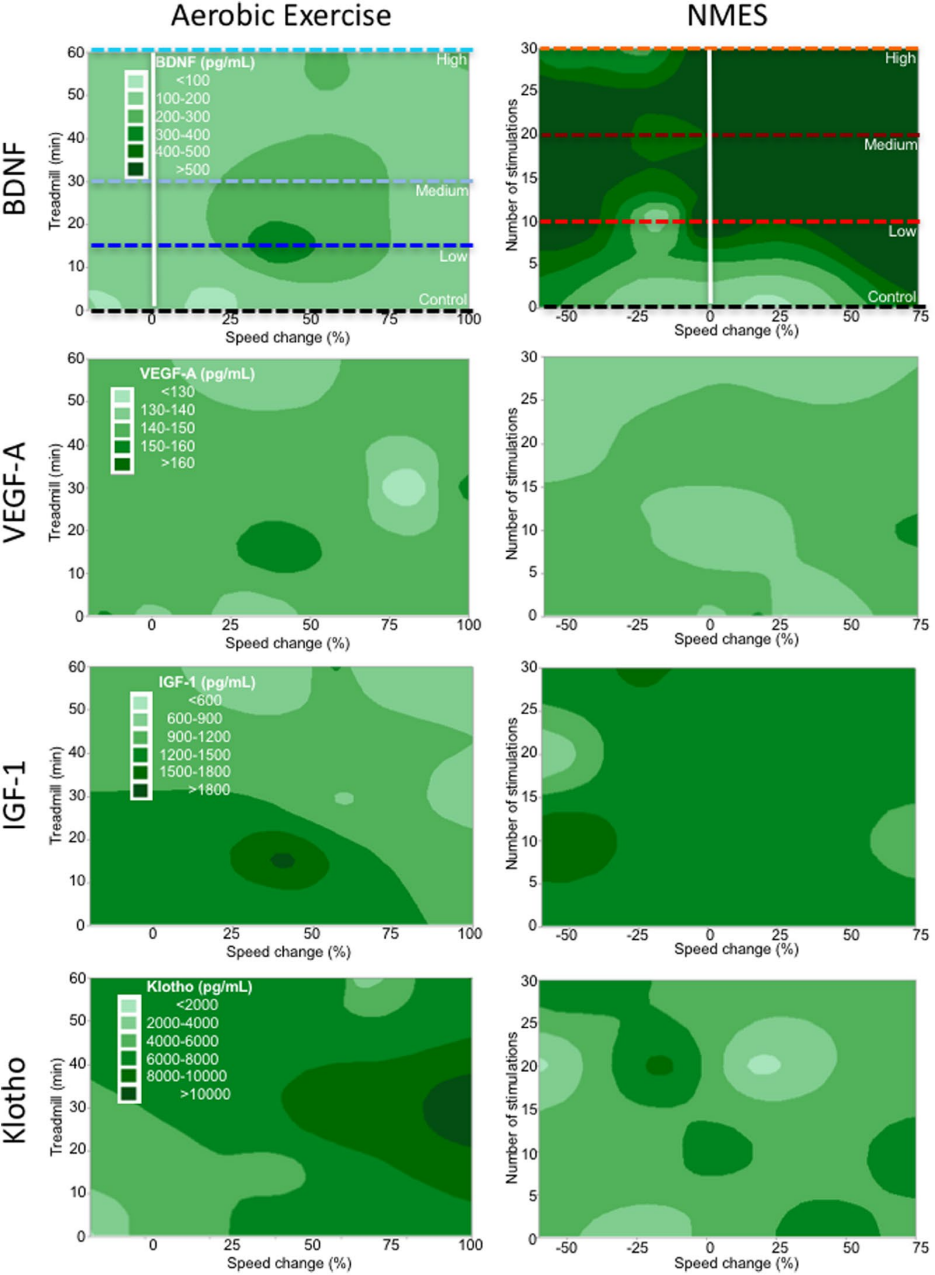
Contour maps – systemic factors. Based on the factorial design that arrayed treatment interventions to a control (0 dose), low, medium and high dose, contour plots of the dependent variables can be drawn to illustrate how these variables interact across the experimental space. It is evident here that systemic BDNF levels were most dramatically increased using NMES, but due to the lack of increase in MCT performance there was little interaction between treatment dose and % change in speed. BDNF did also not reveal a dramatic dose-dependent interaction for AE. A similar lack of interaction was evident for VEGF-A, with some evidence of an increase in levels for a low AE condition that results in a 25–50% change in speed. A clearer interaction between dosing and MCT was evident for AE on IGF-1. A low to medium dose of therapy here produced higher levels of factor release, but this was associated with a poorer performance. Lower levels of IGF-1 were actually induced by the high AE condition, producing the most significant performance change (upper right corner). Minimal interaction was seen for NMES, considering that there was less of an effect on MCT, demonstrating that IGF-1 levels in this paradigm were almost homogenous throughout experimental space. In the AE condition, the contour plot for Klotho indicated that the medium dose produced the highest level of Klotho release, which was associated with the highest level of behavioral change. In contrast the plot for NMES did not reveal a clear interaction between these variables.

To determine if blood biomarkers and behavioral change on the MCT were related, a correlational analysis was performed. Klotho levels were only positively correlated with behavioral changes in the AE condition (r = 0.63, p < 0.05, Table 2). No other measured blood biomarkers revealed significant correlations (Table 2). Contour maps further explored the dosing relationship between treatment conditions and blood biomarkers (Fig. 4). For the AE condition, it is evident that BDNF and VEGF-A are fairly consistent without much interaction between dosing and behavioral change (Fig. 4). In contrast, low doses of IGF-1 were present with a high dose of AE that also produced a high level of behavioral change (Fig. 4). For Klotho, the pattern also reflects changes seen in behavior and dose. The highest levels of Klotho were found with a medium dose of AE inducing the highest behavioral change (Fig. 4). The contour plots for NMES reveal no clear pattern of association between dosing and behavioral change that affects biochemical factors in the blood (Fig. 4). Although it is evident that, for instance, BDNF levels in the blood are very high for NMES with a low dose, this is not associated with a behavioral change (Fig. 4). These results hence further support blood levels of Klotho, and potentially IGF-1, as biomarkers of AE dosing that is also reflective of behavioral changes.

**Table 2:** Correlations between biological variables and MCT.

Assay	Target	Treadmill	NMES
ELISA	BDNF	r = 0.24, n.s.	r = 0.21, n.s.
	VEGF-A	r = 0.03, n.s.	r = 0.09, n.s.
	IGF-1	**r = −0.46, p = 0.1**	r = −0.05, n.s.
	Klotho	**r = 0.63, p < 0.05**	r = 0.17,n.s.
mRNA	BDNF	**r = 0.58, p < 0.01**	r = −0.08, n.s.
	VEGF-A	r = 0.32, n.s.	r = −0.03, n.s.
	IGF-1	**r = 0.73,p < 0.01**	r = −0.08, n.s.
	Klotho	**r = 0.47, p = 0.08**	r = 0.06, n.s.

### Hippocampal BDNF levels reflect NMES dosing

**Figure 5:**
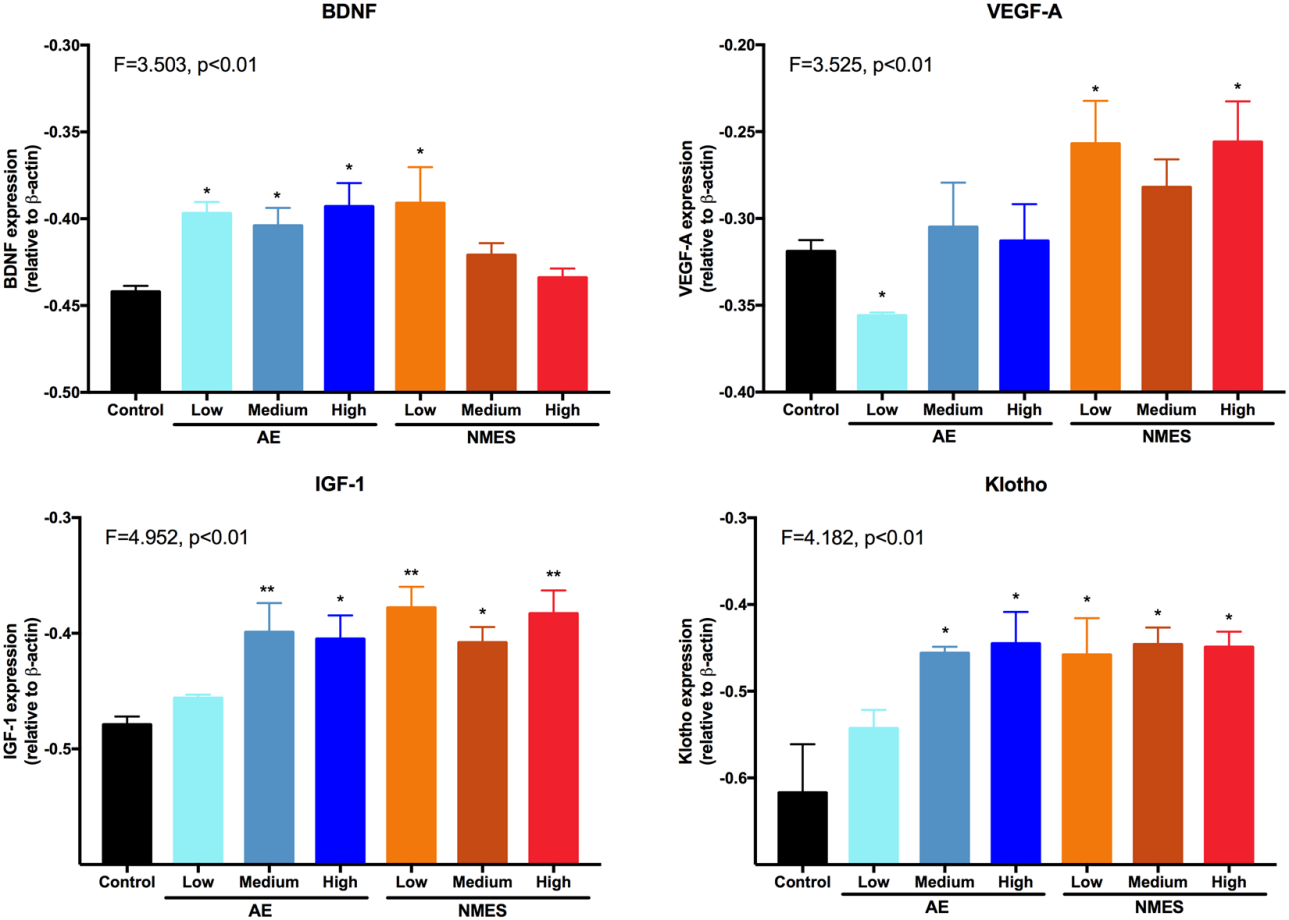
Hippocampal gene expression. BDNF mRNA was upregulated after AE, but did not exhibit a dose-dependency. A significant 10% increase in hippocampal BDNF was evident after low NMES and showed a dose dependent decrease to the level of controls. VEGF-A was upregulated in all NMES conditions, but did not change after a medium and high dose of AE. A low dose actually decreased VEGF-A expression. IGF-1 was upregulated in all conditions by approx. 17%. Only the low AE condition produced a lower 5% upregulation. Klotho revealed an expression profile similar to IGF-1 (27% increase), but with a more marked increase in expression in the low AE condition (12%). (*p < 0.05; **p < 0.01).

Signaling of biochemical factors in the brain were investigated by measuring mRNA expression in the hippocampus. AE equivalently increased BDNF mRNA levels at all doses (p < 0.05, Fig. 5). In contrast, NMES saw an inverse dose effect with a lower dose of NMES significantly (p < 0.05) increasing BDNF mRNA levels compared to the high NMES dose (Fig. 5). VEGF-A mRNA levels were only significantly increased in NMES conditions (p < 0.05), but not in the AE groups. A significant decrease (p < 0.05) in VEGF-A mRNA was seen with a low dose of AE. This was inconsistent with the medium and high AE dose, which revealed VEGF-A mRNA levels similar to controls (Fig. 5). Both IGF-1 and Klotho mRNA levels showed dose-dependent expression increases for the AE conditions (Fig. 5). NMES at all doses significantly increased (p < 0.05) expression of IGF-1 and Klotho mRNA levels, but no dose-dependency was evident (Fig. 5). Expression levels of IGF-1 and Klotho in all of the NMES conditions were comparable to the medium and high AE conditions. The low AE condition produced the lowest non-significant increase in gene expression compared to untreated controls for Klotho (12%) and IGF-1 (5%).

**Figure 6:**
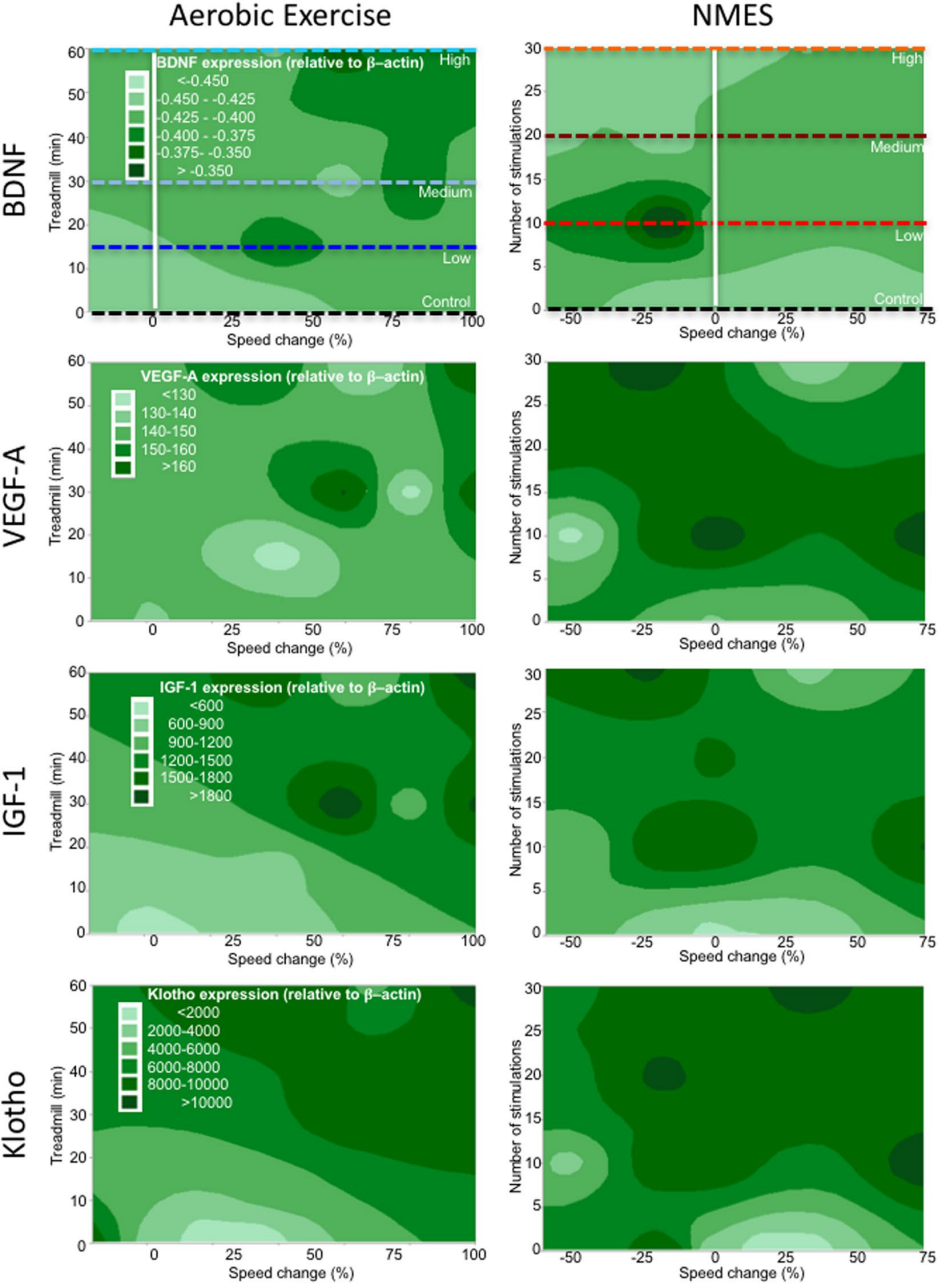
Contour maps – hippocampal gene expression. By accounting for the different levels of dosing for AE and NMES, contour maps were generated to expose interactions between variables in the experimental space. It was evident here that BDNF expression in the hippocampus was highest for the high AE and low NMES conditions. As there was no significant improvement in MCT performance from NMES, this produced a rather flat banding of expression levels. The highest BDNF levels were evident between no change to a 25% performance decrease. For AE, the highest levels were achieved by a high dose that resulted in the highest level of performance increase. VEGF-A revealed the highest increase in the NMES group with a low to a medium dosing leading to peak gene expression. This was associated with improvements in MCT. AE yielded a dose-dependent increase in IGF-1 signaling that was linked to improved performance with a diagonal pattern across the experimental space. A similar effect was evident for Klotho with lower levels being associated with poorer performance and higher levels indicating better performance. A medium dosing of AE is sufficient to achieve peak performance and high levels of Klotho. It was also highly upregulated in the NMES condition with only very high decreases in performance showing a low level of Klotho.

The dose-dependency of IGF-1 mRNA levels was strongly correlated (r = 0.73, p < 0.01) with MCT in the AE condition, whereas Klotho mRNA showed a weaker and non-significant correlation (r = 0.47, p = 0.08). There were no significant correlations between MCT performance in the NMES conditions and hippocampal mRNA expression (Table 2). IGF-1 mRNA expression was highly correlated with Klotho (r = 0.6, p < 0.01) and VEGF-A (r = 0.76, p < 0.001) expression in AE. After NMES, IGF-1 also highly correlated with Klotho (r = 0.63, p < 0.01) and VEGF-A (r = 0.91, p < 0.001) mRNA levels. However, only the IGF-1 blood and hippocampal values in the AE treatment correlated with each other (r = −0.60, p < 0.05, Table 3). This suggests that IGF-1 may be a key signaling factor in mediating performance enhancement after AE. Contour plots further support the role of IGF-1 and Klotho as biomarkers of MCT performance change. AE dose produced an increase in IGF-1 and Klotho, which also related to performance change on the MCT (Fig. 6). NMES contour plots revealed that medium dosing overall produced the most consistent upregulation of mRNA in the hippocampus, but this did not relate to performance enhancements on the MCT. These results hence demonstrate that IGF-1 is a key factor involved in inducing AE-based performance changes and that there is a close coupling to Klotho signaling. It further demonstrates that NMES has effects on hippocampal signaling, but that these are not inducing changes in MCT performance.

**Table 3:** Correlations ELISA and mRNA.

Assay	Target	mRNA	
		Treadmill	NMES
ELISA	BDNF	r = 0.27, n.s.	r = 0.04, n.s.
	VEGF-A	r = 0.09, n.s.	r = −0.04, n.s.
	IGF-1	**r = −0.60, p < 0.05**	r = −0.14, n.s.
	Klotho	r = 0.27, n.s.	r = −0.11, n.s.

## Discussion

The biological and functional effects of different doses of aerobic exercise (AE) and neuromuscular electrical stimulation (NMES) were compared to establish optimal levels of behavioral changes on maximum capacity testing (MCT) and corresponding biomarkers. A dosing effect of AE was evident in MCT with a medium dose providing the most significant gain in performance. This AE dosing was correlated with Klotho changes in blood, as well as IGF-1 and Klotho in the hippocampus. Only blood levels and hippocampal mRNA levels for IGF-1 levels in the AE group revealed a negative correlation. Furthermore, IGF-1 correlated very strongly with VEGF-A and Klotho in the hippocampus, supporting its crucial role in mediating dosing effects in this treatment condition. In contrast, NMES did not significantly affect MCT performance, but revealed significant biochemical changes in the blood and hippocampus. In particular, in the blood BDNF was upregulated in a dose-responsive fashion after NMES, while in the hippocampus an upregulation of VEGF-A, IGF-1 and Klotho was evident. However, there was no evidence of a dose-responsiveness of the expression of these markers. These results therefore provide a direct comparison of AE and NMES on MCT performance, while demonstrating that Klotho and IGF-1 might be key biomarkers of therapeutic dosing.

Although both therapeutic interventions exerted clear biological effects on key biochemical factors in the blood stream and the brain, only AE resulted in an improvement of maximum capacity performance. It is evident here that even a low level of AE improved running performance, but that a medium intensity achieved peak performance, with some decrease evident with the high intensity group. It is important to note here that the aim of using these protocols was not to increase endurance or achieve a maximum performance, as would be the case in athletes^54^, but to evaluate the behavioral effects of commonly used physical therapy paradigms^37^. A variety of AE paradigms have been reported in the literature with little description regarding dosing effects using a functional outcome measure, such as the MCT. However, this is essential to ensure that optimal therapeutic paradigms are employed in both animal and human studies. The treadmill-based MCT described here is a straightforward measure that has translational value considering its routine clinical use. This will facilitate the comparison of rodent models with human clinical studies, which will be indispensable to further advance evidence-based physical therapy interventions and ensure the development of guidelines that are rooted on robustly demonstrated biological changes.

No performance change was evident in any of the NMES conditions, which could be expected considering the lack of involvement of multiple systems (i.e. cardiovascular) in this form of therapy. Nevertheless, some reports indicate that NMES can induce a physiological response akin to that of AE^55–57^, but it remains unclear if this is also producing biochemical and functional changes. It is evident here that IGF-1, VEGF-A and Klotho in the blood were not significantly impacted by NMES. BDNF showed a dose-dependency with the most significant increase being evident with a medium dose of therapy, but these changes did not correlate with MCT, as there was no significant improvement evident on this measure. It is conceivable that performance changes in maximum capacity due to NMES might only be evident in cases of neuromuscular diseases^58^ or after brain injury^42, 52^. Hence, improvements over normal function might not be achievable using this approach, but restoration of performance might be evident in the case of loss of function or in restoring normal muscle tone after overtraining^5, 59^. In severe cases, NMES might be required to improve basic muscle function to facilitate AE. The use of other outcome measures not dependent on aerobic physiology, but dependent on the stimulated muscle groups, might reveal functional improvements after NMES.

In contrast to NMES, AE exerted significant effects on both biochemical factors in the blood, as well as running performance. Although the effects of AE on BDNF, VEGF-A, and IGF-1 have been well documented in the literature^60, 61^, a dose-dependency between blood-levels of these factors and performance changes has not been previously explored in detail. Indeed, BDNF and VEGF-A levels in the blood did not show a correlation with AE dosing. IGF-1 showed a modest correlation, but failed to reach the significance criterion. However, IGF-1 administration has been suggested to mimic the neuroprotective effects of AE and that blockage of its activity abolishes this effect^62^. In contrast, Klotho exhibited a dose-dependency that correlated with performance characteristics and hence provides a better biomarker of AE dosing. Klotho is gradually emerging as an important molecule regulated by skeletal muscle^26^ and is involved in reducing oxidative stress and neurogenesis^63^. Although its interaction with other molecules remains incompletely understood, it is thought to inhibit IGF-1 signaling^64^. and fibroblast growth factors (FGF) signaling^65^.

Complimentary to the systemic effects that biochemical factors released from the muscle exert on the whole body, stimulation of the peripheral nervous system through the muscle provides another pathway to affect localized changes in the brain^66, 67^. Both AE and NMES induced a significant increase in gene expression in the hippocampus. IGF-1 and Klotho revealed an AE dose-dependent increase that was correlated with MCT. IGF-1 was also highly correlated with VEGF-A. IGF-1 was the only factor where blood levels were correlated with gene expression, hence potentially providing a link indicating the influence of systemic effects on brain signaling. It is also worth noting that NMES exhibited a dose-dependent decrease in BDNF levels, with the low NMES condition achieving a high upregulation of BDNF gene expression. However, mRNA and ELISA levels for NMES did not reveal any correlations, potentially indicating that their main mode of action is through peripheral nerve signaling to the brain, rather than the release of factors from the muscle into the blood stream. This would also explain how an increase in BDNF in the blood stream does not correlate with changes in BDNF signaling in the hippocampus. It is also conceivable that other signaling factors in the hippocampus modulate local BDNF expression, which could lead to an uncoupling of BDNF levels from the blood.

The approach presented here provides a well-defined translatable paradigm to investigate the mode (i.e. functional and anatomical changes) and mechanism of action (i.e. molecular changes) of interventions employed in physical therapy. The use of animal models affords the harvesting of brain tissue to investigate local gene expression and to correlate this with performance characteristics, as well as changes in other organs, such as the blood. We demonstrated that a dose-dependent effect can be established for biochemical factors in the blood, as well as the brain. Klotho emerged as a putative blood biomarker of AE, as it correlated with dose-dependent performance changes. However, the key molecule bridging signaling in the blood and the brain is IGF-1, with levels in both organ systems being correlated. IGF-1 was also a central signaling molecule in the hippocampus with it being correlated with VEGF-A and Klotho. Establishing biomarkers of these interventions in normal animals provides a consistent baseline to explore how these factors change against the backdrop of signaling changes in pathological cases, such as musculoskeletal atrophy, acute brain injuries or neurodegeneration. Ultimately, this approach can aid in the design of more efficient treatment strategies, including the identification of optimal frequency and intensity of rehabilitation protocols.

## Electronic supplementary material

Supplementary Figure 1

Supplementary Figure 2
