# Supplementary material for: Biological effects of dosing aerobic exercise and neuromuscular electrical stimulation in rats
Source: Sci Rep. 2017 Sep 7;7:10830. doi: 10.1038/s41598-017-11260-7 (PMC5589775; doi:10.1038/s41598-017-11260-7)
Supplement: Supplementary file 1 — Supplementary Figure 1 [file 41598_2017_11260_MOESM1_ESM.pdf]

Biological effects of dosing aerobic exercise and neuromuscular electrical stimulation in rats.

Stefania Dalise[1,6*], Loredana Cavalli[1,6*], Harmanvir Ghuman[1,2*], Brendon Wahlberg[3*], Madeline Gerwig[4], Carmelo Chisari[6], Fabrisia Ambrosio[1,2,5], Michel Modo[1,2,3, CA]

University of Pittsburgh, [1]McGowan Institute for Regenerative Medicine, [2]Department of Bioengineering, [3]Department of Radiology, [4]Department of Neuroscience, [5]Department of Physical Medicine and Rehabilitation, Pittsburgh, Pennsylvania, USA; [6] University Hospital of Pisa, Department of Neuroscience, Unit of Neurorehabilitation, Pisa, Italy.

* contributed equally

Corresponding Author:
Dr. Mike Modo
University of Pittsburgh
McGowan Institute for Regenerative Medicine
3025 East Carson St
Pittsburgh, PA 15203
USA
+1 (412) 383 7200
e-mail: mmm154@pitt.edu

Supplementary Figures

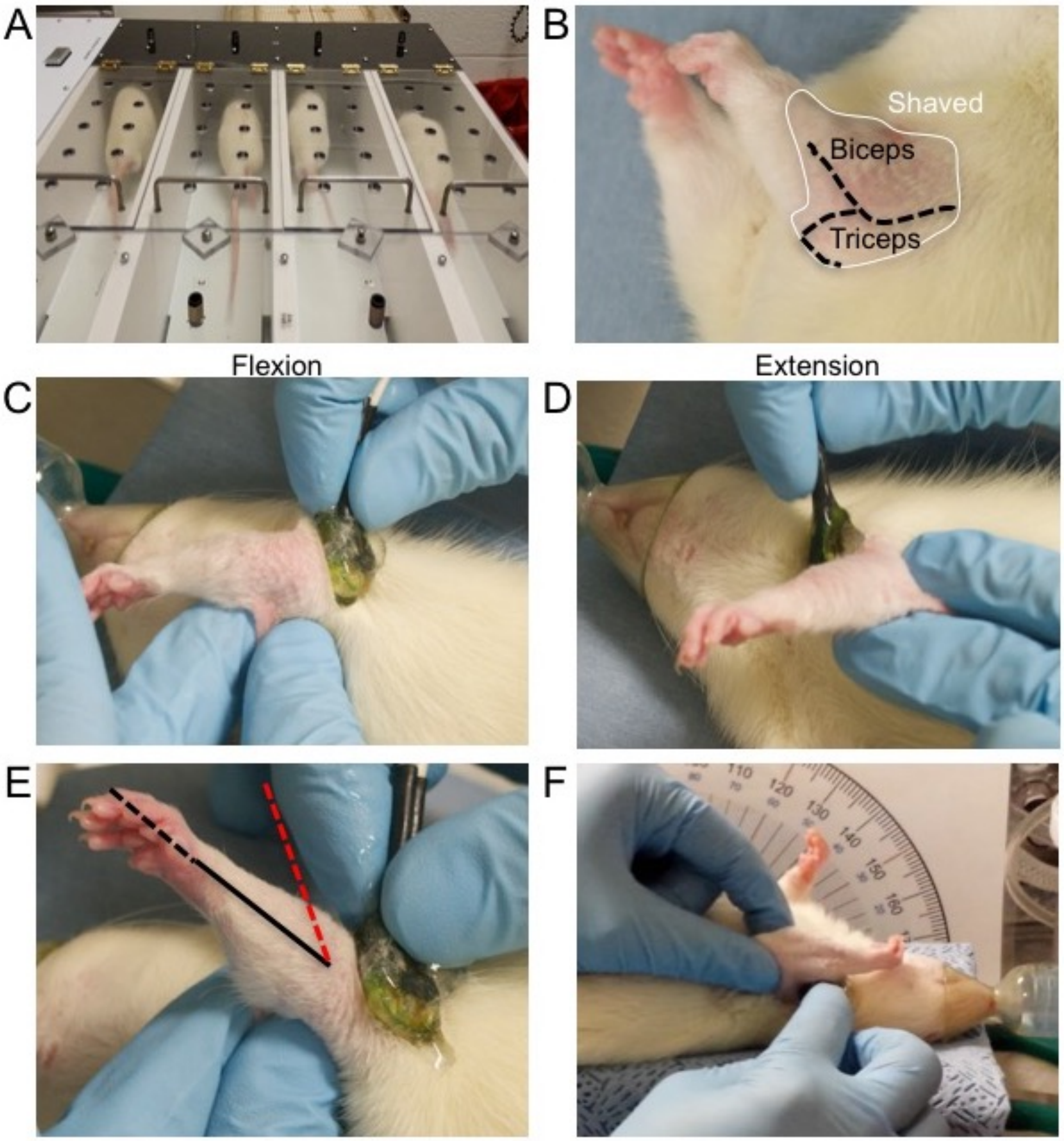

Supplementary Figure 1. Aerobic exercise (AE) and neuromuscular stimulation (NMES). **A**. AE was administered using a treadmill that afforded the running of 4 rats simultaneously. The same apparatus was used to evaluate maximum performance capacity of animals. **B**. For NMES, the biceps and triceps were individually stimulated after exposure of the skin by trimming the overlying hair. **C.** Stimulation of the biceps resulted in a flexion of the forelimb. **D**. In contrast, stimulation of the triceps produced an extension of the forearm. **E.** A 30° extension and flexion of the forearm was considered a successful administration of NMES. **F**. A goniometer was used to determine if an appropriate extension and flexion was achieved.
